# Foreign intervention and legacies in the Ethiopian Orthodox Tewahido Church

**DOI:** 10.1016/j.heliyon.2023.e13790

**Published:** 2023-02-16

**Authors:** Solomon Molla Ademe, Mohammed Seid Ali

**Affiliations:** a Department of Political Science and International Relations, Woldia University, P.O. Box,400, Woldia, Ethiopia; b Department of Political Science and International Studies, Bahir Dar University, Bahir Dar, P.O. Box,78, Ethiopia

**Keywords:** EOTC, Italy, Jesuit missionaries, Legacies

## Abstract

This paper examines foreign intervention and the legacies remain in the affairs of the Ethiopian Orthodox Tewahido Church (EOTC). It discusses the so-called Jesuit missionaries' intervention in the EOTC during the 16th and 17th centuries and some of their legacies. It also explores Italy's intervention in the EOTC in the 19th and 20th centuries and the legacies left in the EOTC. To deal with these issues, this article used a qualitative research approach, including primary and secondary data collection instruments. It shows that contradictory religious teachings, ethnocentrism, and ethnic division, which are evident in the contemporary ecclesiastics of the EOTC, are the legacies of the Jesuit missionaries and Italy in the EOTC. It concludes that the current contradictory and divisive religious teachings in the EOTC were initiated and originated by the Jesuit missionaries, and the ethnocentric tendency and ethnic-based division of top ecclesiastics of the current EOTC are the legacies of Italy. Currently, such divisions are consolidated and celebrated by Ethiopians, including the top authorities of the EOTC, but at least in part, their source is foreign intervention. Thus, the EOTC should disclose the roots of such destructive and divisive legacies to strengthen its unity.

## 1 Introduction

Many foreign countries and individuals interfered in the affairs of the EOTC [1]. Some foreigners criticized and undermined the Ethiopian Orthodox Christians and the EOTC, though many of them admired the church and its laity [1]. The interventions of such foreigners have left legacies in the current EOTC. The foreign interventions that caused the most divisive legacies in the EOTC are the focus of this paper. In this regard, the intrusions of Italy and the Jesuit missionaries are the most vivid cases. Thus, the main objective of this paper is to explore the interventions of the so-called Jesuit missionaries^1^ and Italy in the EOTC in the 16th and 17th centuries and the late 19th and 20th centuries, respectively, and some of their legacies in the contemporary EOTC. This article argues that the legacies left by such foreign interventions have created division and social disharmony among Ethiopians, including the top leaders of the EOTC.

For many writers, politics, religion, and ethnicity are the major causes of the declining national harmony of the current Ethiopian polity. This is true in many cases. But it is important to explore where the roots of this Ethiopian social disharmony lie. For example, Wondimu linked the current Ethiopian social crisis to the ethnicization of the Ethiopian People's Revolutionary Democratic Front (EPRDF) regime [2]. This article does not deny this argument. Instead, it posits that the current Ethiopian crisis should also be viewed from the perspective of foreign interventions and the legacies left in the Ethiopian polity and religion. It mainly focused on the EOTC in order to investigate how foreign interventions left divisive legacies among Ethiopians.

The EOTC was a promoter of Ethiopian unity and the engine of Ethiopia's independence [3]. It is one of the oldest churches in the world and has continuously existed in a close relationship with the Ethiopian monarchy for more than sixteen hundred years [4]. It is a mosaic of many traditions. It shows various cultural influences, including Judaism, African religions, the Syriac tradition, and Coptic Christianity [4]. The Geez script made the EOTC unique. It is the language of the church that contributed to Ethiopia's status as an ancient civilization. It has been able to initiate, celebrate, consolidate, and complete the Ethiopian polity through the Geez civilization. For many years, the patriarchs of the EOTC were ordained among the Egyptians. However, since 1957, the EOTC has been able to appoint an Ethiopian patriarch. The patriarch is the most respected head of the EOTC in Ethiopia and has close ties to political leaders. The patriarchs who ordained until 1974 were appointed through strict religious canons. However, the patriarchs who were appointed after the EPRDF regime were ordained on the basis of ethnicity. The patriarch's appointment based on ethnicity is an anomaly for the EOTC. To make matters worse, currently, for example, some ecclesiastics of the EOTC are declaring the EOTC to be established based on ethnic identities. Some diasporas have already established EOTC churches in the name of their ethnic group. It is against the tradition and the teachings of the EOTC. Thus, dealing with the legacies of foreign intervention in the EOTC is going to have to do with such cases.

Exploring foreign intervention in the EOTC is essential for many reasons. One of the primary advantages of studying such an issue is to identify the legacies that foreign intervention has left in the EOTC. Besides, this article seeks to contribute to our understanding of how foreign legacies become the roots of African socio-political and economic problems, using the EOTC as a litmus test. Indeed, many studies have been conducted on the EOTC. For example, Isaac [5] conducted a study on the history of EOTC. Tamene [6] and Shenk [7] also conducted studies on the EOTC. These authors are some of the dozens of writers on the EOTC. Most of these studies have focused on the history and role of the EOTC in the Ethiopian polity. However, the impact of foreign interference on the EOTC remains understudied.

Excluding the introduction and conclusion, this paper has two parts. The first part discusses the conceptual insights and the research method. The second part deals with the so-called Jesuit missionaries' intervention in the EOTC and its legacies, as well as Italy's intervention and its legacies.

## 2 Conceptual and methodological notes

In the political history of Africa, foreign intervention is mainly associated with colonialism. Obviously, the history of colonialism in Africa is related to Western^2^ states. Western states used various methods to interfere in African states. For example, historically, the civilizing mission was one of the methods [8, 9]. In this regard, Mazrui noted that in earlier years, European colonialism was portrayed as a civilizing force in Africa, Asia, and the non-Western world [10]. The civilizing mission underlined the desirability of reforming and restructuring non-western societies according to the European model [10]. It is what Amin calls “Eurocentrism” [11]. This had implications in terms of potential interference in certain religious customs; if these customs were considered intolerable, it was the moral duty of European Christians and civilized humans to restructure and civilize such an uncivilized society [11]. For Eurocentrists, the other races and cultures appear immature, barbarous, and underdeveloped [12]. Eurocentrists saw their values as the only and highest standard of civilization. For example, European Christianity was considered a measure of civilization. Christianizing the so-called barbarians was one way of carrying out a civilizing mission. Though some countries, such as Ethiopia, are Christian, Europeans regard them as oriental Christians who are far from the rays of modern civilization. In the beginning, Europeans approached the Orient, such as Ethiopia, in the form of peer relations. However, they changed their view of peer Christian nations towards the Orient, believing that they should be modernized in light of European values. In this regard, Ethiopia and the EOTC could be mentioned as the best cases.

There were two steps in which the West could achieve its civilizing mission: rhetoric, or discourse, and action. Firstly, European civilization casts itself as a superior standard [13]. As a result, for the proponents of European civilization, it has become a moral obligation to improve the barbaric, primitive, and coarse races [13]. This is indeed what Ali calls disparagement, or undermining others’ rhetoric [13]. Local perspectives and epistemologies were completely destroyed as a result of such a viewpoint. Second, in order to achieve European civilization or standards, genocidal violence is justified [14]. From the point of view of modernity or a civilizing mission, the barbarian or primitive is in a state of guilt [14]. This allows modernity or European civilization to present itself not only as innocent, but also as a force that will emancipate or redeem its victims from their guilt [14]. It justifies what Teshale Tibebu calls destructive destruction [15]. Thinking about modernity or a civilizing mission might not be a problem in and of itself. The problem arises when the civilizing mission is carried out at the expense of so-called barbarians or “alters.” From the perspective of the alters, the policy was mostly destructive.

The civilizing mission came to an end with the end of formal colonialism. However, western values, policies, and ideologies are continuing even after the end of formal colonialism. Most contemporary African elites, intellectuals, and leaders of institutions view their local epistemology in light of European perspectives. Their view is what Nandy calls “internal colonialism” [16]. It refers to analyzing the values and cultures of a country through a non-native concept or ideology. Such a view is mostly destructive. It subtly causes disorder in indigenous institutions and local values. The intervention of Jesuit missionaries and Italy in the EOTC, as well as their legacies, can show such impacts.

This article used a qualitative research approach. It employed both primary and secondary sources of data. Letters, autobiographies, and reports are among the primary data collected for this study. These sources of data are directly obtained from the owners themselves. The secondary data were also collected from books, dissertations, and journal articles.

In relation to the intrusion of missionaries and their legacies, for example, there are letters that Jesuit missionaries used to write to their principals. Some letters were translated from Latin into English language by scholars. This article used such translated works. In addition to these letters, it used personal biographies written by Catholic monks of the time as additional primary sources. The autobiographies of Alvares and Páez could be listed in this regard. These translated works were used in this article. For example, in relation to Alvares's case, the article used the Amharic document, which was translated by an Ethiopian writer from Portuguese into Amharic. These letters and autobiographies indicated the missionaries' aims and methods for converting Ethiopian Orthodox Christians into the Catholic religion. In other words, the missionaries' messages that we find in these letters clearly show their views on the EOTC.

In relation to Italy's intervention, this article collected data from primary sources such as reports and secondary sources such as books, journal articles, and dissertations. These data were collected from relevant works by scholars in the fields of EOTC, Ethiopian history, and Ethio-Italian history. The study then examines how the current situation in the EOTC is related to the legacies of Jesuit missionaries and Italy. The researcher carefully sorted the relevant works to answer the main objective of the study. The findings were analyzed using thematic analysis in accordance with the objectives of the article. The main limitation of this article is that many of the primary sources were written in non-English languages. However, this limitation was avoided using translated materials.

## 3 The intrusion of missionaries and their legacies

In the second half of the fifteenth and early sixteenth centuries, European powers such as Portugal were more eager than ever to engage with the Christians of the Horn of Africa [17]. Indeed, European travellers, explorers, and envoys had been traveling to Ethiopia before the fifteenth and sixteenth centuries [18]. However, these groups had not brought Europe and Ethiopia into contact. In a state level, the myth of Prester John had played a significant role in the European travel to Ethiopia. Portugal was the first to establish a direct contact between Europe and the mysterious Christian kingdom of the land of Prester John [19]. The main facilitators of Portuguese and Ethiopian relations were the Christian missionaries.

Christian missionaries in Ethiopia had been started in disguise of diplomacy [20]. At the beginning, European elites identified Ethiopia with the distant Christian land of Prester John, the pious king capable of rescuing Christianity from the looming Islamic threat [21]. They assumed that Ethiopia was a powerful Christian country that could be their friend outside of Europe [21]. As a result, some European states, such as Portugal, wanted to have diplomatic relations with Ethiopia. According to some scholars, Portugal was assuming profound advantages in Ethiopia for their actions in helping to save Ethiopian Christians from the Muslim assault. Indeed, the EOTC historians agreed that Portugal's interest was to control Ethiopia. However, this claim seems to be an exaggeration of Portugal's demand. In any case, Portugal was interested in establishing diplomatic relations with Ethiopia. Similarly, Ethiopian Christian kings also made great efforts to establish relations with Europeans, such as Portugal. Then at least through envoys and letter exchanges, Portugal and Ethiopia were able to start diplomatic relations. Ethiopian-Portuguese relation was initially focused on mutual cooperation. For example, in his autobiography, Alvares narrated one of the letters of Emperor Libnedingel^3^ sent to the king of Portugal as:

*I was glad to hear that your father*^4^*had defeated the Muslims. I believe that Christians now have a great protector. I am happy that your father’s messenger has come to tell me that you need to collaborate with me in destroying the Muslims around our kingdoms. Dear Christian Brother, the ambassador to Ethiopia has not yet been sent from a Christian country. The first time I heard about a Christian country like yours was from the messenger who came to Ethiopia with the priests of your country* [22].

The diplomatic relations between Ethiopia and Portugal became strong after the involvement of the Jesuit missionaries in Ethiopia. Before the Jesuit missionaries' intrusion, their relationships were limited to personal exchanges and letters. Including the pre-Jesuit mission, the Jesuits' mission in Ethiopia has four episodes: the pre-Jesuit mission (1520–1544); the first mission of the Jesuits (1557–1577); the second mission of the Jesuits (1603–1622); and the third mission of the Jesuits (1625–1633) [22]. The pre-Jesuit missionaries who entered Ethiopia did not tightly challenge the canons, dogmas, and related values of the EOTC. They had worked hard to bring the Portuguese government into contact with Ethiopia. These missionaries were telling the kings of Portugal, in particular, that Ethiopian Christians were surrounded by Muslim enemies. Ahmad Ibn Ibrihim Al-Ghazi's atrocities against Ethiopian Christians, especially between 1529 and 1540, created a unique situation for Ethiopian-Portuguese diplomatic relations. Ahmad (1506–1543) was the leader of the Adal Muslims. He was able to control most of the Christian empire. Krebs explains that:

*In 1535, at the height of the conflict, dispossessed of most of his realm and in hiding, Libnedingel sent a frantic call for aid to the papacy and Portugal. Among other things, he promised to recognize the pope and enter into a Union of the Churches with Rome in exchange for help* [17].

Despite the delay, Portugal responded positively to the king's call. In the 1540s, four hundred Portuguese fighters arrived in Ethiopia [23]. Pre-Jesuit missionaries contributed a lot to this help. In 1543, the Portuguese-backed Christian Gelawdewos and the Turkish-backed Ahmad fought a final battle at Wayna Daga. In this battle, the Portuguese-backed Christian army defeated Ahmad. After the war, Portuguese troops began to live in Ethiopia. They were also allowed to marry Ethiopian women. Ethiopian monks living abroad were also given a place to live in Rome [24]. Some of the post-war years were happy times for both Ethiopian Christians and the Christian missionaries. However, the euphoria was not sustained for long. The catholic missionaries began to intrude in the affairs of the EOTC. Asires Yenesew explains the post-war condition as follows:

*After the war, a catholic man Bermudez was promoting the Portuguese demand on Ethiopia. Bermudez said to Gelawdewos that he had made an agreement with Gelawdewos’s father.*^5^*According to the agreement, after the victory, half of Ethiopia would be given for Portugal and the EOTC would be dependent on Rome* [24].

Bermudez's question was also what the first Jesuit missionaries asked Emperor Gelawdewos after they entered Ethiopia in 1557. Indeed, Emperor Gelawdewos rejected Bermudez's question (interest) [20]. But this question was not completely closed. The first Jesuit missionaries consolidated the need for the conversion of the EOTC to Catholicism. Indeed, in all episodes, the Jesuit missionaries were aimed at converting the EOTC to Catholicism and making the Roman Church dependent. To facilitate the need for the conversion, the Jesuits set out the attributes of backwardness to the EOTC. Salvadore explains the whole picture of the Society of Jesus on the EOTC as:

*The Society transformed the kingdom of Prester John into a land of mission, articulated a discourse of conquest and attempted to enforce Eurocentric standards. By treating Christian Ethiopians as nonbelievers, the Jesuits fractured Ethio-European commonality and shaped a new discourse of otherness: no longer were Ethiopian Christian brothers to be respected, but rather heretics to be converted* [21].

Some argue that what made these missionaries despise Ethiopian Christians was the Ethiopian kings' repeated request for European help [25]. Ethiopian kings were interested in introducing European technology. Alvares narrated the interest of one of the Ethiopian kings, Libnedingel, as: “I beg you to send me carpenters, printers, producers of all kinds of weapons for warfare, carpenters, pharmacists, doctors, gold and silver refiners, copper mills, and potters [22]. Ethiopian emperors thought that this would be accomplished with the help of the Jesuit missionaries. The missionaries, aware of the Ethiopian emperors’ interest, promised to bring support from the Portuguese government. Bringing European technology was the main tool that the Jesuits used to approach the Ethiopian kings and convert to Catholicism.

Ethiopian kings’ interest of European support created a clear path for the Jesuits to challenge the EOTC. The Catholics who took advantage of this opportunity hastened their role by pretending to be friends with the Ethiopian monarchs. Considering the Ethiopian social structure, the Jesuits used a number of methods to convert the royal family to the Catholic religion. They began to publicly speak and consider Ethiopian Christians as heretics and backward.

In particular, as some second and third Jesuit missionaries indicated through their letters and notes, they agreed that the EOTC was heretical, backward (uncivilized), and uneducated. For example, in a letter that Father Nicolaus de Fenal wrote to the Superior General of the Society of Jesus, he noted that Ethiopian Orthodox Christians are Christians only in name [26]. The Catholic Jesuits considered those Orthodox Christian priests who challenged their teachings as the greatest heretics in the land [26]. Pedro Páez, who led the second Jesuit mission, criticized the EOTC priests and educated people as backward and unmodern [27, 28]. Besides, Páez's successor, Alfonso Mendez, who led the third Jesuit mission and arrived in Ethiopia in January 1624, proved to be less tolerant of Ethiopian Orthodox practices, and he soon proclaimed the primacy of the Roman Catholic Church while condemning local Ethiopian Orthodox Christian practices [29]. In general, the way Catholics view the EOTC is based on their own beliefs and culture. When they saw the EOTC, they realized it was backward and wrong. As a result, they worked tirelessly to convert Ethiopian Christians to Catholicism. The first method of converting Ethiopian Christians to Catholicism was to persuade and convert the royal family. It included a discourse of despising the practices of the EOTC. The second was the last solution, which they employed when the first method failed—it was force to convert Orthodox Christians to Catholicism.

The first method was almost successful. It was the method of Ignatius Loyola, the founder of the Jesuit community. The Jesuits were able to convert some royal families to Catholicism, saying that they would help the Ethiopian kings by bringing support from the Portuguese government [24]. Some Ethiopian orthodox Christians are said to have been voluntarily changed. Later, the main royal families were converted to Catholicism. Some court officials publicly requested the EOTC to be renewed and reformed [30]. A letter of the Gorgora (Gondar) Jesuit monks, which was written in 1609 to the superior general of the Society of Jesuits, says:

*The king embraces us with constant affection, favouring us with goodwill and guarding sedulously against the violence of the monks, and is disposed most excellently toward our faith. We observe the same among the noblemen and ladies, as well as among all of the king’s noble daughters, and whenever we have a dispute with nobles or monastic leaders, with two or three exceptions they are always brought to condemn their own pronouncements as errors* [26].

The conversion of the emperor and his royal family to Catholicism was caused by their expectation of receiving Portuguese support. These emperors were encouraged and warned by the Catholic missionaries. For example, one of the heads of the missionaries, Father Páez, warned Susneyos that “the officials of Portugal will not help you if they do not know whether the EOTC is Catholic. The Portuguese government will assist you if you convert the EOTC to Catholicism [25].”

The Catholic missionaries themselves had shown that Ethiopian kings and the royal families were not converted to Catholicism in faith. For example, an excerpt from the Annual Letter of the Province of Goa (1612) noted Seela Krestos's reason for conversion. As indicated in this letter, the Jesuits do note that the Ethiopians' reasons for converting were not all spiritual. Seela Krstos made it clear that his country wished to obtain military support from the Portuguese government [26]. Seela Krstos was one of the king's top officials. Since 1607, Emperor Susneyos and some noblemen showed increasing sympathy for Catholicism [31]. The king's brother and top official, Seela Krstos, also embraced the new faith in 1613, and helped to spread a Catholic mission in Ethiopia. The king ordered a public debate to be held in order to show his support for Catholicism. This was a debate between Orthodox and Catholic scholars. It was said that during these debates, the emperor was siding with the Catholics. Therefore, the Jesuits expected that they would inevitably be victorious over the Orthodox scholars.

Of all the Catholic patriarchs dispatched to Ethiopia, Páez (1603–1622) was undeniably the most effective in attracting the Ethiopian elites towards Catholicism [21]. In 1621, Emperor Susneyos was converted to Catholicism. He then received Holy Communion from the Jesuit father in 1622 [31]. In 1624, the king declared the Catholic Church as the official religion of Ethiopia [25]. This was the first time the EOTC lost its authority. But after Emperor Susneyos declared Catholic as the country's religion, a popular uprising broke out. The reason for the revolt was the need to restore EOTC as a state religion by discarding the Catholic religion. During this time, the Catholics forced the king to use force. The Jesuits also called on the Portuguese government and bishops in Lisbon and Rome to support the king in his engagement against the rebels. For example, in 1629, Father Mendes wrote a letter to the Roman Pontiff to get gifts for Emperor Susneyos. Accordingly, Mendes asks that the Pope might send a letter of encouragement to the emperor in courageously undertaking war for the sake of the faith [26]. At this time, the Jesuits agreed that military intervention should be the only method to convert the EOTC [21]. Consequently, many EOTC monks had been killed since the emperor was declared Catholic. For example, in one battle in 1632, the armies of the king killed eight thousand Ethiopians opposed to him [4]. Many Orthodox Church monks, including the pope, were executed by the order of the emperor and Catholics. But the massacre did not convert Ethiopian Christians to Catholicism. Instead, the massacre was the end of the Jesuits' stay in Ethiopia. It also ended the reign of King Susneyos.

Fasiledes, the son of Susneyos, came to power in 1932, and the Jesuit mission in Ethiopia was terminated. The first act of the new king was to expel the Jesuits [32]. In 1632, the Ethiopian Orthodox Church reverted to being the state religion. But the Jesuit mission is remembered with real bitterness by Ethiopian Orthodox Christians to this day [4]. Indeed, the Jesuits’ mission was not the only bitter memory. Instead, their divisive legacy is still causing problems in the EOTC.

The bloody war of the era of the princes, which lasted from 1769 to 1855, was one of history's most heinous consequences of Catholic missionaries' legacies. During the era of the princes (1769–1855), religious conflict within the EOTC was often used as a pretext for the powerful rulers to enter war. The religious doctrinal contradiction or differences was one of the traditions introduced by the Jesuit missionaries. In other words, the doctrinal difference was emerged after the teachings of the Jesuit missionaries.

After the teachings of the Jesuit missionaries, the ecclesiastics of the EOTC and royal families were faced with doctrinal schism and confusion. As a result of the Jesuit teachings, Qubat (anointing) was the official doctrine of the Gojjam province; Yetsegalej (son of grace) was the tradition of Gondar and Shewa provinces; and Tewahido (Monophysite) was the doctrine of Tigray and Lasta provinces. Tsegga (son of grace) expresses the view that Christ has undergone three births: the eternal birth of the Son from the Father; the genetic birth of the Son from the Virgin Mary; and the birth by the Holy Spirit during baptism [29]. Qebat (anointing) states that Jesus became a perfect man and a perfect God by the anointing of the Holy Spirit in the Jordan River and not by the incarnation [29]. Monophysite holds the tradition that acknowledges the transformation or absorption of humanity into divinity (also before the Resurrection). It accepted the pre-Chalcedonian tradition [33]. Clearly, Qubat and Tsega traditions were planted after the intrusion of Jesuits into Ethiopia. Before the intrusion of the Jesuits, in all the above-mentioned provinces, Tewahido (Monophysite) was their only religious doctrine. These differences caused the chaos of the era of the princes. Indeed, such differences had been discarded by Emperors Tewodros II (1855–1868) and Yohannes IV (1872–1899). Accordingly, all Christians were told to support the Tewahdo doctrine, the old Orthodox Christian dogmas that came from the Alexandrian church. But these doctrinal differences were not buried. They are still creating divisions in the EOTC today.

Currently, the EOTC is challenged by the legacies of the teachings of the Jesuit missionaries. For example, presently, Tehadiso (the Reformation) and Qubat are the main groups that are creating doctrinal divisions in the EOTC. Proponents of the Tehadiso argue that the EOTC is deviated from the Bible [34]. For example, they argue that the EOTC honours the Blessed Virgin or St. Mary more than she deserves to be honoured. For them, this is heretical. Besides, this group argues that the EOTC is unmodern and backward in many ways. They demand that the whole system of the EOTC needs to be reformed and renewed. For example, Wolde Rufael, the former head of one of the EOTC churches in Addis Ababa, argues that the EOTC needs to renew itself, recognizing that the pace of the world is changing [35]. He claims that the EOTC is not engaged in modern or civilized activities. For the opponents of Tehadiso or proponents of the old tradition of the EOTC, the arguments of this group are closely related to the teachings of the Jesuit missionaries. According to the views of this group, the views of Tehadiso adherents are to undermine the EOTC's tradition by promoting a Catholic tradition and citing the same old argument. In other words, their idea is to undermine Ethiopia's ancient tradition of the EOTC by endorsing the borrowed culture. Even terms such as “uncivilized,” “unmodern,” “backward,” and other terms used by Tehadiso supporters to criticize the EOTC are the legacies of the missionaries. These terms were used by missionaries to criticize the EOTC and its priests.

The other group is Qubat, and this group is a direct descendant of the teachings of Jesuit missionaries. Anointers are within the EOTC. They are even found in some of the EOTC's largest monasteries. Some have even gotten the position of the papacy. For example, there are many anointment believers in East Gojjam. But they do not publicly claim that they oppose the doctrine of the EOTC. Qubats are often seen trying to create doctrinal differences in the EOTC. This again creates a conflict between the main church (EOTC) and the anointers.

The supporters of *Tewahido* criticize these two groups (the supporters of Tehadiso and Qubat) as heretical and adherents of foreign religion. Tewahido believers and scholars argue that the old EOTC should continue to be unaltered. But they argue that some structural practices of the EOTC should be improved. The EOTC is continuing with the wave of these groups. Up to this date, Tewahido has triumphed against the teachings of the adherents of Qubat and Tehadiso. But the EOTC could not dry up such divisions, which are partly the legacies of the intrusion of missionaries.

## 4 The Italian intrusion and its legacies

Ethiopian rulers were eager to establish diplomatic relations with European powers. In particular, those kings who ruled Ethiopia after 1270 wanted to have diplomatic relations with the Christian Europeans. Most of these kings, including Emperor Tewodros, who initiated the modern Ethiopian state, considered Europeans as their best allies [32,36] In the views of these Ethiopian kings, Christianity was the first requirement for establishing diplomatic relations with Europe [37]. Indeed, Europeans were not apathetic towards the diplomatic interests of the Ethiopian kings. As explained in the above section, in the 16th and 17th centuries, through the active involvement of the Catholic missionaries, Portugal was able to establish diplomatic relations with Ethiopia. Besides, in the 19th and 20th centuries, Britain and Italy established diplomatic relations with Ethiopia. According to Asires Yenesew, the interest of Britain and Italy in having diplomatic relations with Ethiopia is linked to colonialism [24]. To prove this, he mentioned Italy at the forefront [24]. Italy was the perfect example of what came to the land of Ethiopia first in political diplomacy and then in a conventional war. Italy's involvement in the Ethiopian polity negatively affected the political, economic, social, and cultural aspects of Ethiopians [37]. From the social and cultural aspects, the EOTC was the supreme example, which was impacted by the Italian invasion and its legacies.

In Ethiopian political history, Italian-Ethiopian diplomatic relations are linked to King Menelik II of Shewa [38]. Indeed, Menelik's treaties with Italy before he became the emperor (king of kings) of Ethiopia confirmed this evidence [38]. Italy's policy of maintaining a diplomatic relationship with Menelik II was to incite Menelik II to act against Emperor Yohannes IV, who ruled Ethiopia from 1871 to 1889 [32]. Asires Yenesew argued that the Italian-Menelik diplomatic relationship was dangerous for both Menelik and Yohannes IV [24]. On the one hand, the treaty between the two partners weakened the power of Yohannes IV, and on the other hand, Italy itself declared war on Menelik II due to this treaty. The Italy-Menelik relationship harmed both Yohannes IV and Menelik II. Diplomatic relations between Ethiopia and Italy, therefore, began before Menelik II became emperor^6^ of Ethiopia. Menelik's contacts with Italy can thus be traced back to 1876. Bahru summed up the diplomatic relationship and the serious threat that the two partners' treaty brought to Ethiopia as follows:

*It was with the coming of Count Pietro Antonelli in 1882, however, that the rather sporadic contacts assumed a deeper and more sustained character. It was to be Antonelli’s fate to preside over both the climax and the nadir of Italy’s relations with Menilek II. His brainchild, the Treaty of Wuchale (1889), was to mark the diplomatic watershed of his career, marking at the same time the high point of friendship between the two parties and the beginning of the hostilities that inexorably led to Ref.* [32].

Italy desired that Menelik remain impartial in their dispute with Emperor Yohannes IV. In other words, Italy did not want Menelik to support Yohannes. After Italy's conquest of Massawa in 1885, there were several skirmishes between Italian soldiers and Emperor Yohannese's armies. Emperor Yohannes IV tried to resolve their conflict peacefully, but his strategy was not accepted by Italy [39]. Indeed, Italy wanted to colonize Ethiopia. To make matters worse, Italy began to expand its presence on the mainland of Ethiopia. As a result, in 1887, Italian troops and Yohannese's troops fought the bloodiest war. The Ethiopian army, led by Ras Alula, defeated the Italians at Dogali [40].

After Dogali, debates in the chamber of deputies in Italy were deeply emotional and patriotic, liberally sprinkled with references to honour and revenge [39]. Various Italian organizations and citizens urged the government to exact vengeance against Ethiopia for the Dogali heroes. These opinions indicated that Italy would invade Ethiopia. Surprisingly, Emperor Yohannes did not think that Italy would invade Ethiopia [41]. The Italians knew that they could not capture Ethiopia if King Menelik supported Yohannes IV. This forced Italy to approach King Menelik of Shoa more than ever. Italy managed Menelik in two ways: by promising guns and diplomatic support to King Menelik and by making treaties with him (King Menelik). The first tactic is linked to material support. For example, Menelik was promised 5000 rifles from Italy for not cooperating with the emperor, Yohannes IV [39]. Indeed, it was Menelik's real reason for negotiating with Italy [39]. Undeniably, Italy's main strategy in colonizing Ethiopia was dividing and weakening Ethiopians [38]. The separation of Menelik from Emperor Yohannes IV was part of this strategy. To undermine Ethiopian unity, Italy employed a ‘provincial divide’ strategy. This divisive tactic made Italy almost successful.

The second tactic that was used by Italy to colonize Ethiopia was to make treaties with Menelik. These include the 1889 Wuchale Treaty. The document of the treaty was initiated in 1888, before the death of Emperor Yohannes. In other words, it was written before Menelik became Ethiopia's King of Kings. The purpose of the treaty was to make Menelik the King of the Kings of Ethiopia and to secure Italy's economic, political, and social interests in Ethiopia. There are 20 articles in this treaty. In particular, Article 17 clearly states Italian colonial interests. Bahru stated that:

*The height of Italian colonial ambition was expressed in the Italian version of Article XVII, which bound Menilek to make all his foreign contacts through the agency of Italy, thereby reducing Ethiopia to the status of an Italian protectorate.* (*The Amharic version had made the use of the services of Italy optional*) [32].

Menelik did not feel bound by the treaty to use the Italian government for his diplomatic affairs. Two things happened before Menelik signed the treaty. The first was the defeat and death of Yohannes IV due to the war with the Dervishes (Sudan) in 1889, and the second was the issue of King Menelik's emperorship. This means that Emperor Yohannes was defeated in the war with the Dervishes without having to start a major war with Italy. Following this, Menelik became the emperor of Ethiopia. Then, Menelik signed the treaty to get Italian support. Antonelli, the Italian envoy, began to shape Menelik around Italian interests. But Menelik was not a simple and foolish leader. Menelik II regarded Italy as a dangerous country [41]. Then, Menelik II entered into a contradiction with his best ally, Italy. To make matters worse, Menelik unilaterally rejected the Italian version of the Wuchale treaty [42]. Bahru describes:

*Antonelli and other emissaries tried hard but in vain to persuade Menilek to accept the Italian version of Article XVII of the Treaty of Wuchale. His abrogation of the treaty in February 1893 dashed the last hope of the Italians to achieve their objective without resorting to arms* [32].

Menelik's decision was unthinkable for Italy. As a result of Menelik's strong rejection of Italy's colonial interests, the Italians were persuaded that their policy of colonizing Ethiopia would fail. Finally, they resorted to force. In March 1896, Menelik's army and the Italian army fought their battle at Adwa. In this war, Italy was defeated by Ethiopia. This victory became a beacon of freedom and emancipation for Africans abroad and made Ethiopians known to the world [43]. The victory of Ethiopia over Italy attracted the attention of the world's media at the time.

This war had great diplomatic and psychological benefits for Ethiopia. Ethiopia had become the pride of Africa [44]. To use Raymond Jonas's words, Ethiopia had gotten the economy of celebrity through Adwa [43]. Italy, on the other hand, was portrayed as a disgrace to white people. So, the war became a source of deep resentment for the Italians.

However, following Ethiopia's victory, many European countries began to enter into diplomatic agreements with Ethiopia. Italy was one of them. For example, in October 1896, Italy and Ethiopia signed the Addis Ababa treaty. This treaty abrogated the much-maligned Treaty of Wuchale, thus marking Italy's recognition of the absolute independence of Ethiopia [29]. Italy also did not show any opposition to Ethiopia's bid to join the League of Nations in 1923. Instead, through its diplomat, Bonin Longare, Italy showed its strong support for Ethiopia becoming a member of the League of Nations. On September 28, 1923, Ethiopia became a member of the League of Nations [45]. In this regard, Nadew Abawalom, who was following the Ethiopian question from Geneva, told the Ethiopian government on Telegram: ‘We have no other reason than to thank the God that made Italy one of the countries that supported and helped Ethiopia to become a member of the League of Nations [45].’ Besides, in 1924, after Fascist Mussolini came to power, Emperor Haile Selassie visited Italy [45]. During the visit, the two countries had talks on peace, brotherhood, and cooperation (Ibid). Mussolini sought to protect Italy's diplomatic interests in Ethiopia. The peak of rapprochement between the two countries was reached with the signing of the Treaty of Peace and Friendship on August 2, 1928 [32]. The diplomatic relations between Italy and Ethiopia in the post-1920s looked like the pre-Adwa treaties. After Adwa, much of Italian-Ethiopian relations were based on mutual benefit and brotherhood. In fact, the 1924 secret treaty of Italy with Britain indicated that Italy was planning to colonize Ethiopia under the guise of diplomacy. The agreement was that for Britain's interest, the two (Italy and Britain) agreed to build a dam on Lake Tana; and for Italy's interest, the two powers agreed to build a road that could connect the Italian colonies of Eritrea and Somalia with Ethiopia [46]. Indeed, both countries apologized to Ethiopia, saying that the agreement violated its sovereignty. But the apology was verbal, not sincere. Italy could not dispel its resentment against Ethiopia. The restoration of Italian glory was, indeed, one of the plans of the Fascist Mussolini. As a result, Italy prepared to invade Ethiopia in the second round. The invasion was a revenge attack for Adwa ([46]. On October 3, 1935, Mussolini spoke: ‘We have endured Ethiopia for 40 years. That's enough [47].’ ‘Mussolini's remarks showed that Italy was dreaming of capturing Ethiopia, even though it was too late to do that [47].’

The immediate cause of the Italian invasion of the 1930s was the Wal-Wal incident. Wal-Wal was a place located between Italian Somali land and Ethiopia. But the main reason was Italy's desire to colonize Ethiopia. This was evident in her actions. When Italy invaded Ethiopia again in the 1930s, it mainly used two tactics. The first was rhetoric, and the second was the end of the first or last resort, i.e., war. The rhetoric presented Ethiopia as an uncivilized country that should be modernized by the intervention of the Europeans. One of the points Italy explained to the world to show that Ethiopia was an uncivilized country was the presence of ethnic oppression in Ethiopia. According to this propaganda, Ethiopians were oppressed by one ethnic group. This tactic was the bitterest Italian way of dividing Ethiopians. Margery Perham stated that:

*Condemnation of Ethiopian slavery had been a lurid theme in Italian propaganda leading up to war and the aggressors seem to have thought that it might have some value internally in Ethiopia if it could be used to drive a wedge between the classes* [48].

Italy explained that this is a sign of the lack of European civilization. This way is very toxic and is still costing Ethiopians. The Amhara and their institutions were identified by Italy as oppressors of other Ethiopians [49]. Italy clearly provoked ethnic hatred among Ethiopians [50]. In other words, a sense of ethnic difference in Ethiopia was created by Italy. Italy also incited Muslims against Orthodox Christians [50]. It clearly followed the policy of diminishing the prestige and authority of the Orthodox Christian Amharas [51]. It also advertised their role in freeing Muslims from oppression [48]. Among the religious institutions, the EOTC was identified as the oppressor institution [50]. To be true, the main method used by Italy to control Ethiopia was weakening the EOTC and Amharas, which they had most to fear. It encouraged Muslims and non-Amhara ethnic groups to weaken the Amhara and EOTC resistance [52].

Italy used a number of spies, writers, agents, religious people (including Ethiopian Christians), and missionaries to facilitate its rhetoric. Among the diplomats and spies, the leading advocates of Italian colonialist discourse were Baron Roman Prochazka, a Czech jurist who arrived in Ethiopia in the late 1920s. He wrote a book under the title “Ethiopia: The Powder Barrel” in 1935, after one year of forceful expulsion from Ethiopia by the order of the government because of his racist behaviour. In his book, he justified Western imperialism in Ethiopia as a necessary and legitimate policy. He argued that “the opponent of imperialism should bear in mind that the numerous non-Amhara native tribes in Ethiopia, which constitute by far the greater part of the total population of the empire, are themselves the victims of Abyssinian imperialism” [53]. He also added that Ethiopians are oppressed by Amharas, who should not be called Christians like Europeans [53]. Ethiopian Christians, according to Prochazka, are barbarians with a mix of heathen and Christian culture [53]. Besides, some Ethiopian-educated Christians such as Afewerk Gebreyesus argued that Ethiopians should be colonized by Italy in order for European modernity to be introduced into Ethiopia. Indeed, Afewerk did not support full-scale Italian colonialism. Instead, he was supporting the transfer of European civilization to Ethiopia through colonialism. In addition to these agents and other pro-Italians, Italy also worked to divide Ethiopians through letters and leaflets. For example, during the war, Italy wrote a letter to the people of Tigray:

*We have been sent by God to expand civilization and provide prosperity to your nation. However, due to the Amhara oppressors, bandits, and bloodthirsty troops, we are unable to carry out this honourable and God-given job of civilizing you. Respected people of Tigray, if you find these Amhara bandits roaming around your village, we urge you to drive them out of your village like dogs without giving them food and water by using your guns. Expel these Amhara bandits if they come to your villages to buy food or drink. If you feed them, you will receive harsh punishment from us* [3].

As a result, the Italians were able to attract the attention of some Tigrian elites. Similar letters had also been disseminated to the ethnic groups living in the southern parts of Ethiopia to prevent their cooperation with the Amharas. In doing so, Italy had attracted the attention of some southern Ethiopian elites. For example, according to Ezekiel Gebissa, the Oromo elites were showing cooperation with the Italian colonialists [54]. These Oromo elites began to feel that they had been oppressed by the Amharas and orthodox Christians. So, the rhetoric used by Italy to invade Ethiopia was successful.

After using such rhetoric, Italy used a second method, i.e., war. Destruction was the last resort that Italy used to defeat Ethiopia. This was what Western imperialists call a “just war” [13]. War was the ultimate solution to subjugating and civilizing the barbarians [13]. Italy also used to destroy the so-called barbarians (the EOTC and Amharas) in such a way. Surprisingly, most Europeans did not oppose this step when Italy declared war on Ethiopia [55]. Even most military commentators at that time were against the Ethiopians. For example, neither the use of gas nor the employment of aircraft against civilians was seen as taboo or created significant outrage among military observers. Instead, they lauded the Italians' steady logistical efforts and employment of artillery and airpower to overcome nature and the enemy's resistance [55]. When Italy was about to win and after it won Ethiopia, Italy weakened the Amhara and the EOTC to end the question of Ethiopian independence. For example, it declared Arabic as an official language for Ethiopia, except for the Amhara provinces, as a way of weakening the Geez script and Amharic language [51].

However, neither the EOTC nor the Amharas stopped their resistance against Italian colonialism. This forced the Italians to justify the genocidal war against the EOTC and the Amharas. Italy killed many monks, bishops, and Ethiopian Orthodox Christians, whom it assumed were against the Italian invasion. For example, in 1937, over 2000 monks were massacred at Debre Libanos Monastery by the order of the Italian viceroy in Ethiopia, Rudolf Graziani [49]. Including one of the four bishops of the EOTC, Abune Petros, many priests and monks were killed in the early days of occupation, including the monks who were in the monastery of Zukwala, Gondar, and other places [48]. It also burned nearly 2000 EOTC churches [50]. To make matters worse, the Italians became the makers of the canons and guidelines of the EOTC [50]. Above all, Italy has left destructive legacies in the EOTC. The Italian intervention took place many years ago. But the rhetoric that Italy constructed in the EOTC continues to be inherited by the present generation.

Ethiopian ethnic elites and leaders of different institutions today have reintroduced Italian rhetoric and discourse. The obsession with ethnicity in the EOTC today is a litmus test that can show the continuation of the Italian legacy. Just as Italy portrayed the EOTC as an Amhara institution, some ethnic nationalists still view the EOTC as an Amhara institution [56]. Just as the Italians burned down many EOTC churches, claiming that the EOTC was an Amhara institution, some extremist nationalists are still burning the churches of the EOTC for the same cause [56]. Just as the Italians killed the bishops of the EOTC, contemporary Ethiopian politicians killed religious leaders of the EOTC. In the same way that the Italians appointed EOTC patriarchs in violation of the EOTC's canons, contemporary Ethiopian political leaders did the same in the modern EOTC. For example, both post-1991 patriarchs were appointed under the intervention of the Tigray-led government. The first post-1992 patriarch was Father Paulos, and his appointment brought another split. It separated the holy synod of the EOTC. After the death of Abune Paulos, Matias, who also came from the Tigre ethnic group, was said to have been appointed with the help of the Tigray-led government. As a result, some Amhara EOTC monks in Gondar and Gojjam blamed the appointment process of Father Matias [57]. They opposed the Synod of the EOTC because of Father Matias's strong affiliation with the then regime.

Recently, ethnicity has been propagated and promoted, even by some top leaders of the EOTC. In 2019, for example, a group led by priest Belay advocated for the formation of a separate Oromo Orthodox Tewahido Church [58]. This movement was supported by many ethnic Oromos. They believe that the Oromo people should use their own language within church services, including liturgy and preaching. Basically, from a democratic point of view, their question seems correct and logical. The main point to be underscored here is that the intention behind establishing the Oromo EOTC is to play their part in dividing and dissolving the EOTC, not just church services in Afan Oromo. That was the main reason the Synod condemned the movement. In addition, the need to establish the EOTC on the basis of ethnicity seems to have been accepted by some Tigrayan church leaders as well. For example, some Tigrians in the West have named their churches in their ethnic names. A perfect example of this is the establishment of the Debre Selam Kidist Silassie Tigrian Orthodox Tewahido Church in Philadelphia [59]. In addition to the Tigrian diasporas, in the beginning of 2022, Tigrian diocese of the EOTC declared that they suspended their subordination to the EOTC [60]. Surprisingly, Tigrians are among the ancient Orthodox Tewahido believers. They are the ones who have brought the EOTC to its current level. The establishment of the Tigray Orthodox Tewahido Church in isolation from the mother church (EOTC) seems to be a disgrace to the history of the people of Tigray. Some Tigrian church leaders, however, are advocating for the separation of the Tigrians from the EOTC. It is one of the worst legacies of Italy's divisive rhetoric.

## 5 Conclusion

This article explored the intrusion of foreigners into the EOTC and attempted to demonstrate the legacies they left behind. Specifically, it explored the intrusions and legacies of the Jesuit missionaries and Italy in the 16th–17th and 19th–20th centuries, respectively. It argues that the intrusion of these foreigners into the EOTC has left divisive legacies in the current EOTC. These legacies are still affecting the EOTC.

Jesuit missionaries’ legacies, for example, contributed to the doctrinal differences in the present EOTC. Similar discourses and actions that the Jesuit missionaries used to blame and undermine the EOTC are still seen in the EOTC. Just as the Jesuit missionaries did, opponents of the EOTC, such as Tehadiso supporters, still argue that the EOTC is uncivilized, unmodern, and does not follow the path of the time. And, for its part, the EOTC condemns its opponents as heretics, nostalgic for other cultures, and materialistic (unspiritual). Currently, though it is not hot, such debates and problems are becoming common within the EOTC.

The Italian intrusion also left ethnic divisions in the contemporary EOTC. These legacies are undermining the unity of the EOTC. This means that there is a fear that the EOTC may be divided along ethnic lines. Some of the fears are surfacing on the ground. For example, in 2019, some Oromo priests in the EOTC mobilized to establish an independent Oromo Orthodox Church. In 2021, though it was a decision made without the recognition and will of the synod of the EOTC, the Diocese of Tigray declared the establishment of an independent Tigray Orthodox Church in Tigray. These examples point to the dire situation in which the EOTC can be divided on ethnic lines.

It is clear that these divisive problems are wreaking havoc on the EOTC. The EOTC can bring unity in a variety of ways. One of them is examining the sources of such divisions. If EOTC and its followers examined the roots of divisions, they could at least identify where the problem lies. Understanding the source of the problem can be part of the solution. Thus, the EOTC should disclose the roots of such destructive and divisive legacies to strengthen its unity. As a result, this article suggests further research that tries to shed light on how to avoid such divisive legacies.

## Author contribution statement

Solomon Molla Ademe; Mohammed Seid Ali: Conceived and designed the experiments; Performed the experiments; Analyzed and interpreted the data; Contributed reagents, materials, analysis tools or data; Wrote the paper.

## Funding statement

This research did not receive any specific grant from funding agencies in the public, commercial, or not-for-profit sectors.

## Data availability statement

No data was used for the research described in the article.

## Declaration of interest's statement

The authors declare no conflict of interest.

## Additional information

No additional information is available for this paper.

## Footnotes

1 The missionaries who came to Ethiopia in the 16th and 17th centuries were given the name “Portugal Missionaries” by most Ethiopians. But it is clear that these missionaries were also Spanish and Italian citizens. Indeed, even the Ethiopian emperors of that time knew that they were Portuguese missionaries. In the academic literature, these missionaries are commonly known as Jesuit missionaries. This paper acknowledged this term and used it in the section that explored Jesuit missionaries. The center of these missionaries was Rome.

2 In this article, the west (western states) are the so-called imperialist powers, which took part in colonialism.

3 Libnedingel was the king of Ethiopia (from 1508 to 1540).

4 Your father refers to the king of Portugal who was succeeded by his son.

5 Emperor Gelawdewos's (r.1540-1559) father (predecessor) was emperor Libnedingel (r.1507-1540).

6 In the Ethiopian political system, the king is under the emperor (king of kings) and accountable to the latter. For example, when Menelik was the king of Shewa, he was accountable to the emperor of Ethiopia, Emperor Yohannes IV.
